# Practice of Pre-Hospital Emergency Care and Associated Factors in Addis Ababa, Ethiopia: Facility-Based Cross-Sectional Study Design

**DOI:** 10.2147/OAEM.S424814

**Published:** 2023-09-06

**Authors:** Azanaw Abebe, Zegeye Kebede, Dereje Bayissa Demissie

**Affiliations:** 1 Schools of Nursing, St. Paul’s Hospital Millennium Medical College, Addis Ababa, Ethiopia

**Keywords:** pre-hospital healthcare providers practice

## Abstract

**Background:**

Medical emergencies require quick field interventions and stabilization before transport, while rapid transportation to definitive healthcare with fewer field interventions improves trauma outcomes. Poor prehospital healthcare practices negatively impact patients’ health, and limited studies exist on providers’ practices in resource-limited areas like Ethiopia. This study aimed to assess the practice of pre-hospital emergency care and associated factors at pre-hospital health facilities in Addis Ababa, Ethiopia.

**Methods:**

A facility-based cross-sectional study was conducted 191 pre-hospital healthcare providers, of which 20 randomly selected participants were participated in the observational study from October 2021 to February 2022 in Addis Ababa Ethiopia. Data was collected using a checklist and self-administered questionnaire. Data was cleaned, entered into Epi data version 4.4, and exported to SPSS software for analysis. Binary and multivariable logistic regression analyses were performed, with a P-value of 0.05 considered statistically significant.

**Results:**

The study found that 43% (82) of pre-hospital healthcare providers in Addis Ababa, Ethiopia, had good practice in pre-hospital emergency care. The identified factors that increased the odds of good practice in pre-hospital emergency care were: being able to provide advanced life support (AOR = 88.99; 95% CI: 27.143–291.603); adequate monitoring and defibrillators (AOR = 5.829; 95% CI: 1.430–23.765); having work experience of 4–5 years (AOR = 5.86; 95% CI: 1.424–24.109); and having the opportunity to continue education (AOR = 31.953; 95% 6.479–157.591).

**Conclusions and Recommendations:**

The study found high levels of poor practice among pre-hospital healthcare providers in Addis Ababa, Ethiopia. Factors contributing to good practice include being trained in advanced Life Support, adequate monitoring, defibrillators, work experience, and having the opportunity to continue education. Therefore, policymakers and health planners should establish teaching and training centres based on Ministry of Health and Education guidelines.

## Plain Language Summary

Medical emergencies, such as cardiac arrests, benefit more from quick field interventions and stabilization before transport, while rapid transportation to definitive healthcare with fewer field interventions by trained paramedic professionals improves trauma outcomes. A facility-based cross-sectional study was conducted from October 2021 to February 2022, with 191 prehospital healthcare providers working in prehospital healthcare facilities in Addis Ababa. Participant observational study data were collected using a checklist, followed by a self-administered questionnaire administered by trained data collectors at each prehospital health facility. The identified factors increased the odds of good practice of pre-hospital emergency care, including advanced life support, adequate monitoring and defibrillator, and 4–5 years of work experience and opportunities to continue education.

More than half of the prehospital healthcare providers had poor prehospital care practices. Factors that increase the likelihood of good practice include competently performing ALS, adequate monitoring and defibrillation, work experience, and opportunities for continuing education. Policymakers and health planners should establish prehospital healthcare teaching and training centers based on prehospital healthcare guidelines.

## Introduction

This article examines ED visit rates and crowding in 15 countries outside the United States. Emergency department (ED) visit rates have increased, causing ED crowding, a public health issue. Supply and demand mismatches cause long waiting times, delays in critical treatments, and higher complication rates and mortality. This study suggested that understanding causes and potential solutions can help mitigate ED crowding in the US and other countries around the World.^1^

An emergency medical service (EMS) is the approach rendered to a patient at the scene and during transport before the initiation of in-hospital patient care and is designed to rapidly treat and transport seriously ill or injured patients to the Emergency Department (ED).^2^,^3^

Pre-hospital emergency care is medical care provided to patients before arrival at the hospital after the activation of the emergency team. Pre-Hospital Care refers to emergency medical services rendered to an emergency patient for analytic, resuscitative, stabilizing, or preventative purposes before transfer to healthcare facilities.^4^,^5^

Pre-hospital workers should transport a severely injured victim from the scene of the injury to definitive care at a fixed facility as quickly and safely as possible. In Mexico, poor selection of medications and late referral to hospitals were judged to be important contributors to more than half of deaths. An increase in the number of ambulance dispatch sites reduces deaths among patients route to hospitals.^6^,^7^ The study in Sri Lanka shows; the required skills for advanced life support, most of the staff showed skills in IV cannulation (71.4%) and IV drug administration (71.4%) however skills were shown in cricothyroidotomy (22.9%), pleural drainage (25.7%) and laryngoscopy and intubation (31.4%).^8^

A study in Ethiopia showed that trauma outcomes are better managed by rapid transportation to definitive health care and fewer field interventions, whereas medical emergencies, such as cardiac arrests, benefit more from prompt field interventions and stabilization before transport.^9^

Another study in Gondar, Ethiopia, showed that the majority of participants knew about basic life support (76.2%) and how cardiopulmonary resuscitation (CPR) (77.0%) was performed. More than half of the patients received adequate basic life support and cardiopulmonary resuscitation (CPR). Less than half of the participants (42.3%) had training related to pre-hospital emergency care.^4^

A study in the United States showed that 32% of paramedics reported having no experience as paramedics before providing critical care.^10^ A previous study conducted in Mexico and Japan reported that poor selection of medications and late referral to the hospital were important contributors to more than half of the deaths, and the willingness to perform CPR among health students before BLS training was 13% respectively.^11–13^ A study in England showed that the most common assessments recorded by paramedics were respiratory (20.1%), pain (15.8%), and deceased (7.9%); 74.4% were transported to the hospital (99.5%). Among those with pain as the primary impression, (53.9%) received analgesic.^14^

In a qualitative study in India, most trauma victims in Delhi were rapidly brought to the hospital by bystanders, taxis, and police and sometimes died on the way. However, ambulances are common and primarily used for interfaculty transfer.^15^ A study in England showed that 60–78% of study participants did not practice the clinical skills of first aid management of heat and heat illnesses, burns, shock, sprains and fractures, poisoning, and road traffic accidents. Forty% of the participants did not practice the clinical skills of recording respiratory rate and temperature.^16^ A qualitative study conducted in Qatar showed that, during transportation, patients were exposed to potential risks related to falling on stretchers, medication errors, and clinical judgment.^17^

A study in Tanzania showed that higher education levels and first-aid training were associated with higher practical knowledge scores.^18^ About 91.5% of patients stay in EDs for over 24 hours due to inadequacy of beds, overcrowding, and delays in radiological services. Organizational commitment is crucial to providing sufficient admission beds, scaling up laboratory test profiles, and decreasing radiology service turnaround time to improve LOS and patient transportation practices.^19^ In Gondar, Ethiopia, approximately 41.5% of emergency workers, including pre-hospital health providers, have inadequate basic life support (BLS) and 50.1% have inadequate advanced life support (ALS).^4^ The poor practice among pre-hospital healthcare professionals results in compromised patient care, transportation, and safety.^6^,^11^ Pre-hospital healthcare providers are the first healthcare workers to reach a patient who needs special attention, regular training, and well-organized infrastructure. This study is important for policymakers, prehospital healthcare providers, and researchers to organize training systems, good practices, and education systems periodically. The Ethiopian Emergency Medical Services Protocol Guideline (FMOH) requires adjusting ambulance bed height to ensure safe patient movement and secure all patients, especially children, using appropriate straps, carriers, or immobilization devices. This study, the first of its kind in Ethiopia, serves as a baseline for future research and will help improve prehospital healthcare professionals’ knowledge and practice in patient transportation.

## Methods and Materials

### Study Setting

The study was conducted in Addis Ababa City Administration, and the pre-hospital health facilities found in Addis Ababa were Addis Ababa Fire Emergency Prevention and Rescue Agency (FEPRA) was established at 1933 G.C in Arada sub-city, which has nine dispatch branches with three-digit call numbers (939) and works with more than 165 healthcare providers.^20^

The Ethiopian Red Cross Society (ERCS) Addis Ababa branch has nine nurses working as pre-hospital healthcare providers, and the website ambulance pre-hospital emergency medical service is the first private pre-hospital emergency medical service providing company in Ethiopia has 17 EMTs and nurses A total of three pre-hospital healthcare facilities in Addis Ababa with total of 191 pre-hospital healthcare providers registered and working as pre-hospital healthcare providers in Addis Ababa, Ethiopia.

Study design: A facility-based cross-sectional study was conducted between October 2021 and February 2022. Sample size determination and Sampling procedures.

The sample size was calculated using a single population proportion of practice of prehospital healthcare providers, which was 0.5,^4^ 95% confidence interval, and the margin of error was taken 0.05 rate =5%. The initial sample size was 384. All pre-hospital healthcare providers who had registered and worked as pre-hospital healthcare providers in Addis Ababa, Ethiopia in mid of 2022 were included.

The sample size was corrected using a formal correction of a finite population. The calculated sample size for the first objective was 141, whereas the calculated and maximum sample sizes for the second objective were 164. Therefore, the maximum sample size was 164. However, the population size was 191, which was a manageable total census of prehospital healthcare providers, and the final required sample size was 191. A total of 191 prehospital healthcare providers working in prehospital healthcare facilities in Addis Ababa were included, of which 20 were included in the observational study using standardized checklists.

### Data Collection Tools and Data Collection Procedure

To ensure the quality of data collection, the questionnaires were adapted after different studies were reviewed based on the research objectives, and the checklist questionnaire was adapted and modified based on the BLS and ALS guidelines. The equipment availability questionnaire was adapted and developed based on the national minimum requirement for ambulance services (FMHACA, Ethiopia).

Data were collected using a structured, self-administered questionnaire and an observational checklist. Data was collected by five trained paramedics professionals. The data collection training was given for two days which was included practical sessions. Training and orientation about the objectives and relevance of the study on each item included in the study tools and the entire process of data collection were provided to data collectors and supervisors. Regular supervision and follow-up were performed during the data collection. Supervisors checked each questionnaire daily with further cross-checked by the principal investigator for completeness and consistency of data. Prior to data collection pretesting of the data tools were done on 5% of the study population. After the pretest, the questionnaire was checked for its clarity, completeness, reliability, consistency, and sensitivity. Then, corrections were made accordingly. The pretest results indicated that items were managed for internal correlation (Cronbach’s alpha (α) of 0.85) and instrument reliability determined (α=0.79). Then, corrections were made accordingly. Data were collected using an observational checklist by the principal investigator during the data collection period with patient care before self-administered questionnaires were distributed. All observational data were collected from participants who were working on the patient during the data collection period, informed after the observational data were completed, and consent was obtained. Participant observational study data were collected using a checklist, followed by a self-administered questionnaire administered by trained data collectors at each prehospital health facility. Ethiopia’s healthcare structure includes prehospital services, with practitioners like doctors and nurses handling complex procedures like intubation. Basic life support is performed by nurses with on-the-job training, while advanced life support is performed by trained doctors, MD+ specialists, emergency and critical care nurses, and paramedics recertified by the Ministry of Health and Ethiopian Food and Drug Authority (EFDA).

### Operational Definitions and Terms

#### Basic Life Support (BLS)

Basic Life Support (BLS) is non-invasive basic interventions and rapid transport to the definitive health care facility. Interventions are usually basic and include non-invasive CPR, ventilation, and oxygenation, stopping bleeding, immobilization, and manual open airway, positioning patient, lifting, and moving.

#### Advanced Life Support (ALS)

ALS includes all the BLS procedures with the addition of invasive procedures such as endotracheal intubation, intravenous line placement, fluid replacement, and the administration of controlled and potent medications.

#### Emergency Medical Services

EMS are designed to rapidly treat and transport seriously ill or injured patients to the Emergency department.

#### Pre-Hospital Healthcare Providers

Pre-hospital healthcare providers are qualified healthcare providers (can be Nurse, medical doctor, health officer or paramedics) characterized by performing Basic Life Support, Advance Life Support, and lifting and moving patients in the pre-hospital area.

#### Practice of Pre-Hospital Emergency Care

Practice of pre-hospital emergency care was assessed by pre-hospital healthcare providers who scored mean value and above were labelled as good practice, whereas pre-hospital healthcare providers who had scored below the mean were labelled as poor practice.^4^,^21^,^22^

### Data Analysis

Data were cleaned, coded, entered into Epi-data 4.4, and exported to SPSS version 26 for further analysis. Descriptive statistics including tables, figures, and frequency distributions were used to describe the data. Both observations and interviews were descriptively analyzed. Bi-variable and multivariable analyses were conducted using a binary logistic regression model to assess the possible association of independent factors with the practice of good practice of pre-hospital emergency care among health care providers at PV <0.05, and AOR with 95% confidence interval (CI). Finally, the findings are presented in the tables, text, and figures.

## Result

### Socio-Demographic Characteristics

Of 191 prehospital healthcare providers, 178 (94%) were included in this study, with a response rate of 94%. The mean age of the participants was 27.81 with a standard deviation of ±4.012. The majority of the participants were female 95 (53.4%). Of the respondents, 130 (73%) were in the age range of 25–35 followed by ≤ 24 years 41 (23%). About 114 (64%) were single participants and 64 (36%) were married. Concerning the experience of pre-hospital healthcare providers in pre-hospital care, 55 (30.9%) participants had ≥ 6 years of experience, but the majority (77, (43.3%)) had ≤ 3 years of experience, with a mean of 4.48±2.851. Most participants (119, (66.8%) were diploma nurse holders, and the remaining 42 (23.6%), 9 (5.1%), and 8 (4.5%) were degree nurses, EMT, and master’s nurses, respectively. Concerning monthly income, 131 (73.6%) participants had incomes ranging from 3201 to 5250 with a mean of 4983.57±989.99 Ethio-birr. Of the respondents, 152 (85.4%), 17 (9.6%), and 9 (5%) worked on the Addis Ababa fire, Tebita, and Ethiopian Red Cross Addis Ababa branches, respectively. About 126 (70.8%) participants had first aid training, of which 19 (10.7%) participants had first aid training within six months and 52 (29.2%) had never received first aid training (Table 1).

**Table 1:** Socio-Demographic Characteristics of Pre-Hospital Healthcare Providers Working at Addis Ababa Fire, Addis Ababa Red Cross, and Tebita in Addis Ababa, Ethiopia, 2022

Variable	Category	Frequency	Percent (%)
Sex	Male	83	46.6
	Female	95	53.4
Marital status	Single	114	64
	Married	64	36
	Others	0	0
Experience in pre-hospital healthcare	≤ 3years	77	43.3
	5–4years	46	25.8
	≥ 6years	55	30.9
Educational level	Diploma nurses	119	66.8
	Degree nurses	42	23.6
	Emergency Medical Technician (EMT)	9	5.1
	Masters’ nurses	8	4.5
Working site	A.A fire	152	85.4
	Tebita	17	9.6
	Red Cross	9	5
Age category	≤ 24	41	23
	25–35	130	73
	≥ 36	7	4
Monthly income category	3201–5250	131	73.6
	5251–7800	45	25.3
	>7800	2	1.1
First-aid training	Yes	126	70.8
	No	52	29.2
If yes to first-aid training, when?	Before 6 months	107	60.1
	Within 6 months	19	10.7

### Organizational Related Factors

Of the total respondents who had worked ≤ 12 h per day, 97 (54.5%) and the remaining 81 (45.5%) worked 13–24 hours with a mean of 17.37. Of the participants who had the opportunity to continue their education 79 (44.4%). Regarding resource availability, 151 (84.8%) participants said that the work site had good resources, and of 178 respondents, only 26 (14.6%) said that pre-hospital guidelines were available.

### Facility Related Factors

Only 67 (37.6%), 78 (43.8%), and 28 (15.7%) participants worked with adequate drugs, ventilation and airway equipment, monitoring, and defibrillators, respectively. Adequate immobilization devices, bleeding control devices, communication devices, and infection protection devices were used by 173 (97.2%), 177 (99.4%), 155 (87.1%), and 138 (77.5%) patients, respectively.

Practice of pre-hospital emergency care among pre-hospital healthcare providers by participator observational study result reported that less than half (43% (6/14) of the observational activities were performed correctly by the participant. Among 178 participants, 20 participants were included in observational checklist activities Among 20 observed pre-hospital healthcare providers 60%(12/20), 65%(13/20), 75%(15/20), 80%(16/20), 75%(15/20), and 70%(14/20) were open airway, position patient, lifting and moving patient, measure patient blood glucose, stop bleeding and feel pulse rate respectively. Only 30% (6/20), 35%(7/20), 40%(8/20), 15%(3/20), 40%(8/20), 30%(6/20), 10%(2/20), and 40%(8/20) were correctly inserted into the airway, suction airway, ventilation and oxygenation, laryngoscopy and intubation, open IV line, calculated fluid, administered IV drug, and immobilized patient, respectively (Table 2).

**Table 2:** Findings from Participator Observational Study (n = 20) in Addis Ababa Pre-Hospital Healthcare Providers 2022

Variable	Performed correctly	Not Performed correctly
Open airway and check to breathe	12(60%)	8(40%)
Insert airway	6(30%)	14(70%)
Suctioning airway	7(35%)	13(65%)
Ventilate/oxygenate patient	8(40%)	12(60%)
Perform laryngoscopy and endotracheal intubation	3(15%)	17(85%)
Open Intravenous (IV) line	8(40%)	12(60%)
Calculate fluid	6(30%)	14(70%)
Gave Intravenous (IV) drugs	2(10%)	18(90%)
Immobilize patient	8(40%)	12(60%)
Position patient	13(65%)	7(35%)
Lifting and moving patient	15(75%)	5(25%)
Measure patient blood glucose level	16(80%)	4(20%)
Stop bleeding	15(75%)	5(25%)
Feel pulse rate	14(70%)	6(30%)

### Practice of Pre-Hospital Emergency Care

Of the participants, 178 (100%) had experience in lifting and moving patients and of 178 participants 114 (64%) used proper body mechanics during patient lifting and moving. About 123 (69.1%) participants had good communication with co-workers and about 149 (83.7%) participants performed basic life support properly, but only 68 (38.2%) performed advanced life support properly. Approximately 80 (45%) participants received proper quality CPR (Table 3).

**Table 3:** Practice of Pre-Hospital Emergency Care Among Pre-Hospital Healthcare Providers’ Practices in Addis Ababa Fire, the Ethiopian Red Cross Addis Ababa Branch, and Tebita; Addis Ababa, Ethiopia, 2022

Variable	Response	Response in number	Response in Percent
Experience in Lifting and moving patient	Yes	178	100
	No	0	0
Use proper body mechanics	Yes	114	64
	No	64	36
Communicate properly with co-workers	No	55	30.9
	Yes	123	69.1
Perform proper basic life support (BLS)	Yes	149	83.7
	No	29	16.3
Perform properly advanced life support	Yes	68	38.2
	No	110	61.8
Apply quality Cardiopulmonary Resuscitation (CPR) during the patient arrest	Yes	80	45
	No	98	55

**Figure 1:**
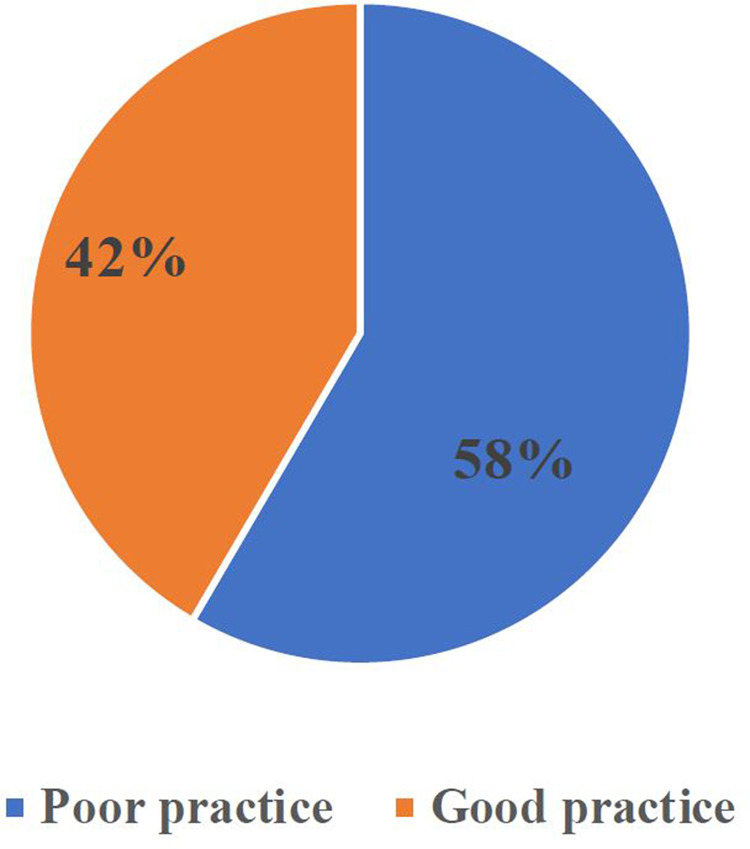
Practice of pre-hospital emergency care at pre-hospital health facilities in Addis Ababa, Ethiopia, 2022.

This study determined that 43% of pre-hospital emergency care providers had good practices (95% CI: 34.8–48.9) (Figure 1).

The study found that less than 50% of pre-hospital emergency care providers had good practice in both observational and self-administered questionnaires, with 43% and 42% having similar levels.

### Factors Associated with Practice of Pre-Hospital Emergency Care Among Pre-Hospital Health Care Providers

After describing the data, bi-variable and multivariable analyses were conducted using a binary logistic regression model to assess the possible association of independent factors with the practice of pre-hospital healthcare providers. Variables with a p-value of ≤0.25 in the bi-variable analysis, were fitted to the final model (multivariable analysis). Variables transferred to the multivariable analysis included sex, first-aid training, performance of ALS, rate of resources, availability of pre-hospital healthcare guidelines, availability of ventilation and airway equipment, availability of monitoring and defibrillator, availability of communication devices, availability of infection protection devices, years of experience with pre-hospital care, and opportunity to continue education (Table 4).

**Table 4:** Identified Factors Affecting Practice of Pre-Hospital Emergency Care Among Pre-Hospital Health Care Providers, Addis Ababa, Ethiopia, 2022

Variables	Category	Practice		p-value	COR (95% CI)	p-value	AOR (95% CI)
		Good	Poor				
Performing properly ALS	Yes	63	5	1	1	1	1
	No	11	99	0.000	0.009(0.003–0.270)	0.000	88.99(27.143–291.60)
Availability monitoring and defibrillator	Yes	21	7	1	1	1	1
	No	53	97	0.000	0.182(0.073–0.456)	0.014	5.829(1.430–23.765)
The opportunity of continuing education	Yes	60	19	1	1	1	1
	No	14	85	0.000	0.052(0.024–0.112)	0.000	31.953(6.479–157.591)
More than one year of work experience with pre-hospital emergency care	<= 3	13	64	1	1	1	1
	4–5	24	22	0.000	0.099(0.044–0.224)	0.014	5.86(1.424–24.109)
	≥6	37	18	0.124	0.531(0.237–1.190)	0.008	6.435(1.639–25.262)

**Abbreviations**: ALS, Advanced Life Support; AOR, Adjusted Odd Ratio; BLS, Basic Life Support; CI, Confidence Interval; COR, Crude Odd Ratio;

A variable passed in multiple variable analysis (p< 0.05) was correctly performing advanced life support, availability of monitoring and defibrillator, opportunity for continuing education, and at least one year of working experience with pre-hospital care. These variables were significantly associated with prehospital healthcare practices. Properly performing advanced life support was 89 times more likely to have good practices than their counterparts (AOR= 88.99; 95% CI: 27.143–291.603). Likewise, a participant who had adequate monitoring and a defibrillator was 5.83 times more likely to have good practice than those who had not (AOR=5.829; 95% CI:1.430–23.765). Moreover, pre-hospital healthcare providers who had 4–5 years of work experience were 5.86 times more good practice than <= 3 years of experience (AOR=5.86; 95% CI:1.424–24.109). The other variable that was significantly associated with good practices in prehospital healthcare was the opportunity to continue education. Pre-hospital healthcare providers who had an opportunity to continue education were revealed to be 32 times more likely to have good practices than their counterparts (AOR=31.953; 95% CI: 6.479–157.591) (Table 4).

## Discussion

This study aimed to determine prehospital healthcare providers’ levels of practice competency in prehospital care and its associated factors. Based on the self-administered practical questionnaire, approximately 42% (CI%:34.8–48.9) of the participants were found to have good practice in pre-hospital care, and based on the observational checklist, it was 43%, which was almost the same, indicating that pre-hospital healthcare providers practice competencies were below 50%. Competent ALS, availability of monitoring and defibrillators, more years of work experience with pre-hospital care, and an opportunity for continuing education were found to be significantly associated with the practice of pre-hospital healthcare. The proportion of good practice among pre-hospital healthcare providers working at Addis Ababa fire and emergency prevention and rescue agencies, Ethiopian Red Cross Society Addis Ababa branch, and Tebita ambulance were found to be 43% with the self-administered questionnaire and observational checklist, respectively. This finding was lower than that of previous studies conducted in Malang, Indonesia (75.4%),^22^ and was also supported by a study conducted in the Free State, South Africa (48.8%),^23^ and some related studies in Gondar, Ethiopia 49.5%.^4^

There are several possible explanations for this discrepancy in the results. First, in Ethiopia, most of the pre-hospital healthcare providers who worked at the pre-hospital site had ≤ 3 years of experience, and most of them were diploma nurses. Additionally, in pre-hospital settings, most pre-hospital healthcare providers received first aid pieces of training for a long time (before 6 months). Moreover, work hours per day were long. This could contribute to the poor practice of pre-hospital emergency care. In the Free State, South Africa, it could be due to job dissatisfaction and a riskier type of job, and in Gondar, Ethiopia, the same health system.

Participants who performed ALS correctly were 89 times more likely to have good practice than their counterparts. This number was significantly higher than that reported in a previous study conducted at the National Hospital, Sri Lanka^8^ and a related study in Gondar, Ethiopia.^4^ This discrepancy may be due to this study being conducted site include a private pre-hospital site (Tebita ambulance services) And participants were pre-hospital providers only unlike Gondar Ethiopia.

Pre-hospital healthcare providers who had an opportunity to continue their education were revealed to be 32 times more likely to have good practice than their counterparts This number was significantly higher than those reported in studies conducted in the free state in South Africa^23^,^24^ and Dar es Salaam, Tanzania.^18^ Possible evidence for this could be geographical and cultural differences.

Another factor independently associated with the practice of pre-hospital healthcare providers in pre-hospital care was work experience. Pre-hospital healthcare providers who had 4–5 years of work experience were 5.86 times more good practice than <= 3 years of experience and. This finding is significantly lower than the study conducted in Malang, Indonesia,^22^ The possible explanation could be because Malang, Indonesia may their pre-hospital system began a long time before Ethiopia-then may have experienced workers and may have adequate resources, the more experience particular works and had adequate resource results from good practice and this study was higher than studies conducted in Gondar, Ethiopia.^4^ This could be because this study was only performed on pre-hospital sites by pre-hospital providers, may have had more pre-hospital training opportunities, and included private sites.

Likewise, the availability of monitoring devices and defibrillators was significantly associated with good practice in prehospital healthcare. participants who were worked at sites of Available monitoring and defibrillator were found to have 5.83 times more likely to have good practice than their counterparts. There have been no previous studies of these factors. A possible explanation could be that the availability of the monitoring and defibrillator helped the participant use it any time they needed and knew the patient’s condition and support to intervene accordingly, resulting from good practice.

In an observational study, only 15% of participants practiced laryngoscopy and intubation. This rate was lower than that reported in a study conducted in a national hospital in Seri Lanka (31.4%).^8^ A possible explanation could be that less exposure to laryngoscopy and intubation resulted in poor practice, and that the Seri Lanka study was performed at a hospital emergency site. The percentage of participants who had the ability to perform IV cannulation and IV administration was 40% and 10%, respectively.

This finding is higher than that of previous studies conducted in national hospitals in Sri Lanka 71.4%.^8^ This discrepancy could be because the study sites were different (hospital vs prehospital).

This study has clinical implications for policymakers and practitioners in developing effective strategies on pre-hospital healthcare providers in Addis Ababa, Ethiopia, had good practice in pre-hospital emergency care and improve emergency treatment outcomes. Also made significant scientific contributions by identifying factors associated with good pre-hospital healthcare providers practice and highlighting the importance of pre-hospital emergency care services.

## Strengths

This study is important for policymakers, prehospital healthcare providers, and researchers to organize training systems, good practices, and education systems periodically. The Ethiopian Emergency Medical Services Protocol Guideline (FMOH) requires adjusting ambulance bed height to ensure safe patient movement and secure all patients, especially children, using appropriate straps, carriers, or immobilization devices. This study, the first of its kind in Ethiopia, serves as a baseline for future research and will help improve prehospital healthcare professionals’ knowledge and practice in patient transportation.

## Limitation

Since the retrospective cross-sectional study does not show a causal relationship and selection bias is not avoidable. Since the study was conducted facility based may limit the generalizability of the result to general populations in the country.

## Conclusion

This study determined that most prehospital healthcare providers’ prehospital care practices were poor. It identified factors that increase the likelihood of good practice by performing ALS properly, availability of resources (monitoring and defibrillator), work experience, and opportunity to continue education as independent predictors of good practice of pre-hospital healthcare providers. Therefore, policymakers and health planners should establish pre-hospital healthcare teaching and training centers based on the pre-hospital healthcare guidelines recommended by the Ministry of Health and the Ministry of Education.

Less than half (43%) of the observed participants practiced prehospital healthcare well. Laryngoscopy and endotracheal intubation, intravenous cannulation, drug administration, fluid calculation, airway insertion, airway suction, patient immobilization, and ventilation and oxygenation were improperly performed during observation. Policymakers and health planners should establish prehospital healthcare teaching and training centers based on the prehospital healthcare guidelines recommended by the Ministry of Health and Education.

## Abbreviations

ALS, Advanced Life Support; AOR, Adjusted Odd Ratio; BLS, Basic Life Support; CI, Confidence Interval; COR, Crude Odd Ratio; CPR, Cardiopulmonary Resuscitation; ED, Emergency Department; EMS, Emergency Medical Services; EMT, Emergency Medical Technician; ERCS, Ethiopian Red Cross society; FEPRA, Fire and Emergency Prevention and Rescue Agency; FMHACA, Food, Medicine and Health Care Administration and Control Authority; FMOH, Federal Ministry of Health; IV, Intravenous; NGO-Nongovernmental organization; PHCP, Pre-hospital Healthcare providers; SPHMMC, Saint Paul’s Hospital Millennium Medical College; WHO, World Health Organization.

## Availability of the Data and Materials

Summary data are available in the main document. All data generated or analyzed in this study are included in this published article.
